# NGS barcoding reveals high resistance of a hyperdiverse chironomid (Diptera) swamp fauna against invasion from adjacent freshwater reservoirs

**DOI:** 10.1186/s12983-018-0276-7

**Published:** 2018-08-14

**Authors:** Bilgenur Baloğlu, Esther Clews, Rudolf Meier

**Affiliations:** 1 Centre for Biodiversity Genomics, University of Guelph, 50 Stone Road E, Guelph, Ontario N1G2W1 Canada; 2 Tropical Marine Science Institute, National University of Singapore, 18 Kent Ridge Road, Block S2S, Singapore, 119222 Singapore; 3 Lee Kong Chian Natural History Museum, 2 Conservatory Drive, Singapore, 117377 Singapore

**Keywords:** NGS barcoding, Tropical streams, Invertebrates, Chironomidae, Community structure, Environmental heterogeneity, Turnover

## Abstract

**Background:**

Macroinvertebrates such as non-biting midges (Chironomidae: Diptera) are important components of freshwater ecosystems. However, they are often neglected in biodiversity and conservation research because invertebrate species richness is difficult and expensive to quantify with traditional methods. We here demonstrate that Next Generation Sequencing barcodes (“NGS barcodes”) can provide relief because they allow for fast and large-scale species-level sorting of large samples at low cost.

**Results:**

We used NGS barcoding to investigate the midge fauna of Singapore’s swamp forest remnant (Nee Soon Swamp Forest). Based on > 14.000 barcoded specimens, we find that the swamp forest maintains an exceptionally rich fauna composed of an observed number of 289 species (estimated 336 species) in a very small area (90 ha). We furthermore barcoded the chironomids from three surrounding reservoirs that are located in close proximity. Although the swamp forest remnant is much smaller than the combined size of the freshwater reservoirs in the study (90 ha vs. > 450 ha), the latter only contains 33 (estimated 61) species. We show that the resistance of the swamp forest species assemblage is high because only 8 of the 314 species are shared despite the close proximity. Moreover, shared species are not very abundant (3% of all specimens). A redundancy analysis revealed that ~ 21% of the compositional variance of midge communities within the swamp forest was explained by a range of variables with conductivity, stream order, stream width, temperature, latitude (flow direction), and year being significant factors influencing community structure. An LME analysis demonstrates that the total species richness decreased with increasing conductivity.

**Conclusion:**

Our study demonstrates that midge diversity of a swamp forest can be so high that it questions global species diversity estimates for Chironomidae, which are an important component of many freshwater ecosystems. We furthermore demonstrate that small and natural habitat remnants can have high species turnover and can be very resistant to the invasion of species from neighboring reservoirs. Lastly, the study shows how NGS barcodes can be used to integrate specimen- and species-rich invertebrate taxa in biodiversity and conservation research.

**Electronic supplementary material:**

The online version of this article (10.1186/s12983-018-0276-7) contains supplementary material, which is available to authorized users.

## Background

Freshwater ecosystems are under threat worldwide from habitat destruction, pollution, and climate change. As a result, global freshwater biodiversity is declining more rapidly than the diversity of many stressed terrestrial ecosystems (e.g., 1–8% species loss per decade: [1]). Such loss of freshwater biodiversity affects food webs, nutrient cycling, climate, air quality, and water supply [2, 3]. One problem with monitoring the health of freshwater systems is the lack of efficient and rapid assessment tools for species-rich invertebrates [4–8] that often constitute much of the biomass and occupy many critical niches. A good example is non-biting midges (Chironomidae: Diptera) that are an important indicator taxon because they are found in most freshwater habitats [5, 9–12], have high specimen abundance, and are particularly species-rich (sometimes having more species than all other insect species in an aquatic environment combined [13]). In addition, the larval stages of chironomids are relatively immobile. Therefore midge communities have the potential to reflect water quality in sampling locations [5, 14]. Chironomids are also an important food source for predators such as odonates, fish, and birds, and act as important decomposers of organic matter [10, 15, 16]. However, reliable sorting/identification to species-level using traditional techniques is so expensive that in many studies chironomids are either only identified to genus/subfamilies, or they are altogether neglected [4].

The cost of midge identification via morphology is high because it usually requires dissection and mounting of specimens onto microscopic slides ([17–19]; i.e., 15–20 min per specimen: [20]). Moreover, it is usually the larvae that are collected while the species names and much of the identification literature is for adults [21, 22]. As a result, species-level chironomid data are rarely used although access to such information would be desirable because different chironomid species vary in their sensitivity to environmental parameters [5, 10, 11, 23–26]. For instance, congeners in *Cricotopus*, *Polypedilum*, and *Tanytarsus* differ considerably with regard to their tolerance to heavy metals, pesticides, and nutrient-levels [27, 28]. It is here that the DNA barcodes obtained with Next Generation Sequencing (“NGS barcodes”) can help because they allow for fast, cost-effective (<USD 0.40/specimen), and thus large-scale species-level sorting with apparently little impact on taxonomic accuracy [6, 20, 29], because DNA barcodes are capable of distinguishing most species of Chironomidae (80–90% congruence: [18, 30, 31] and allow for studying the composition of taxonomically complex chironomid communities [5, 25, 32–35]. NGS barcodes are arguably the next logical step because they overcome the cost problem of traditional “Sanger” barcodes (USD 8–17/specimen: [6, 22, 29]) and allow for barcoding all specimens even if a sample is specimen-rich.

We here use NGS barcodes for > 14.000 chironomids to study the species richness and turnover between adjacent natural and artificial urban habitats. The artificial habitats are three reservoirs (Lower Peirce: 62 ha, Upper Peirce: 303 ha; Upper Seletar Reservoir: 313 ha) while the natural habitat is Singapore’s largest swamp forest remnant (90 ha) which is home to slow-moving and small-sized streams (< 2 m wide, depth < 80 cm: [36]). Note that all three reservoirs have similar environmental conditions [37] due to water transfers [38] and the midge fauna of the reservoirs has been regularly sampled as part of a freshwater quality monitoring program. Combined, the reservoirs are five times larger, and the boundaries are less than 1 km away from the swamp forest [36]. The plant and vertebrate species of this swamp forest have been previously studied, but prior to this study its chironomid fauna was largely unknown [39]. Note that this swamp forest is the largest remnant of its kind in Singapore and thus of high national conservation value. This was also one of the motivations for testing whether its chironomid fauna is resistant against the anthropogenically-mediated biotic influences of the adjacent reservoirs.

By studying midges from reservoirs and the swamp forest, we hope to contribute to a better understanding of chironomid species turnover in tropical habitats. More specifically, we first quantify the species diversity of the chironomid fauna in the swamp forest remnant using NGS barcoding applied to a large specimen sample. The second aim is to compare the chironomid fauna of the adjacent urban and natural habitats. The replacement of native with urban species can lead to undesirable homogeneous biotic communities by diminishing the faunal distinctions between habitats and regions [40]. As shown for some taxa in urban-gradient studies (plants; [41], ants; [42, 43], birds; [44]), native species are being replaced with urban species upon the invasion of natural habitats. However, there is very little data for invertebrates and even less for chironomids. Much of the midge research focuses on nuisance species while their impacts on the adjacent native fauna have received less attention [15, 45–47]. The third aim of our study is to understand species turnover within the swamp forest communities. We use the available environmental information to study the correlation between community composition and these parameters via multivariate statistical analyses. We specifically ask what environmental variables determine the chironomid community in the swamp forest, whether any species are intermixing between the habitats, and if so, whether the urban reservoir species invade the adjacent wild habitats?

## Methods

### Field sampling

#### Swamp forest – Sampling larvae

Between October 2013 and December 2014, 40 sites in the slow-moving streams of Nee Soon Swamp Forest were sampled (see Additional file 1: Table S1) by the Tropical Marine Science Institute (TMSI). These sites, located within the protected Central Catchment Nature Reserve (CCNR), were selected to represent the whole catchment. CCNR covers 20 km^2^ and is surrounded by highways and major roads as well as residential areas. For each sampling site, 12 physical and chemical parameters (cross-sectional area, stream width, stream order, stream velocity, stream discharge, maximum depth, average depth, turbidity, dissolved oxygen, and pH) were collected, and GPS coordinates were recorded (see Additional file 2: Table S2 for details). As the freshwater streams in Singapore are short, narrow and shallow (i.e., ranging from 1 to 2 m width and 10–80 cm depth) [48], qualitative kick sampling as described in [49] was used at each site, where chironomid larvae were collected using kick nets (36 × 30 cm, 250 μm mesh size) over a 2-min period along three replicates of 10 m stretches. All larvae (*n* = 6620) were preserved in isopropanol.

#### Swamp forest – Sampling adults

As part of a long-term insect biodiversity project, one site (1°23′00.3″N 103°48′46.5″E) in the deep forested segments of Nee Soon Swamp Forest was sampled for adults using two Malaise traps between January 2012 and January 2013, four times a month. Alcohol-preserved adult specimens (*n* = 1551) were extracted from these samples.

#### Reservoirs

The midge samples came from Lower Peirce (62 ha, 7 m depth), Upper Peirce (303 ha, 22 m depth), and Upper Seletar Reservoir (313 ha, 17 m depth) [37, 38] and were sampled as part of freshwater quality monitoring. The samples were collected from Upper Seletar using an Ekman grab measuring 20 cm × 20 cm and from Lower and Upper Peirce Reservoirs using stainless steel cages, i.e., colonization-type invertebrate sampler, measuring 20 cm × 10 cm, described in Loke et al. [50]. The colonization samplers were designed for Singapore’s aquatic habitats to enable invertebrate collection from hard-bottomed urban reservoirs [50, 51]. The specimens were preserved in isopropanol. We here include those samples that were collected during the same time periods that were covered by the swamp forest survey. They are Upper Seletar (*n* = 3647: October 2013 to June 2014, 11 sampling dates), Upper Peirce (*n* = 1056: January to April 2014, three sampling dates), and Lower Peirce (*n* = 1306; January to April 2014, three sampling dates). Environmental variables were not collected for the reservoirs. Therefore, the reservoir chironomids were only used for species diversity and turnover analysis.

### PCR amplification and NGS barcoding

NGS barcodes were amplified for each specimen using the direct polymerase chain reaction (direct PCR) protocol described in [20] that avoids DNA extraction. PCR reactions were carried out in 20 μL volumes containing 2 μL of BioReady rTaq 10× Buffer, 1.5 μL of 2 mM dNTP mixture, 0.25 μL of BioReady rTaq DNA polymerase, 2 μL (1 mg/mL) of BSA and 2 μL of 10 uM forward and reverse primers. Specimen-specific amplicon sequencing was carried out using unique combinations of tagged primers ([29], Baloğlu et al., unpublished). Degenerate metazoan primers (COI; mlCO1intF: 5’-GGWACWGGWTGAACWGTWTAYCCYCC-3′ [52] and jgHCO2198: 5’-TAIACYTCIGGRTGICCRAARAAYCA-3′ [53]) were used for the new PCR reaction conditions. The samples that failed at direct PCR stage were processed with QuickExtract (Quick Extract DNA™). The specimens were immersed in 20 μl of the extraction solution and otherwise processed following the manufacturer’s instructions. PCR products were pooled and sent for library preparation. NGS barcoding of specimens (*n* = 14.180) was carried out on multiple MiSeq 2 × 300 cycle runs that also sequenced specimens for other projects.

### MOTU delimitation

Sequences were delimited into molecular operational taxonomic units (MOTUs) using Objective Clustering at 3–5% with uncorrected pairwise distances [54]. This range of thresholds has been shown to produce a stable number of clusters that is largely congruent with species boundaries as determined by morphology ([29], Baloğlu et al., unpublished). Some of the resulting MOTUs could be identified to species using an available barcode database for midges that was generated from specimens which were identified to species based on morphology as part of a nuisance midge study [19].

### MOTU identification

In order to determine whether a barcode pertains to a midge species, we use two checks. The first is based on morphology and consists of two steps. The samples were first presorted by parataxonomists with experience in processing biomonitoring samples. Second, each specimen was then again handled individually during the direct PCR setup; i.e., morphologically disparate specimens unlikely to belong to Chironomidae were eliminated. However, it can be difficult to distinguish chironomid larvae from the larvae of close relatives such as Ceratopogonidae [55]. We, therefore, implemented an additional quality control step at the genetic level. Each haplotype was BLASTED against Genbank’s COI database (accessed in October 2017) using MEGABLAST and identifications were obtained using Readsidentifier [56]. The results were used to eliminate barcodes that may not pertain to Chironomidae. We kept all barcodes that satisfied one or several of the following criteria (see Additional file 3): (1) barcode match to Chironomidae > 96% (39 MOTUs). (2) Top 10 BLAST hits pertaining to Chironomidae (229 MOTUs). (3) 7–9 of the top 10 BLAST hits are Chironomidae (4) < 7 of the top 10 BLAST hits are Chironomidae, but the remaining hits are to very different taxa (15 MOTUs: hits to Tachinidae, Drosophilidae, Syrphidae, Muscidae, moth, etc.). (5) MOTUs with > 10 specimens with all top hits to Schizophora. These were kept because Schizophora larvae cannot be confused with midge larvae and the large number of specimens rules out pre-sorting error (5 MOTUs). (6) Lastly, we kept those MOTUs (*N* = 8) where some of the top hits were to other aquatic Diptera, but the midge hits had higher identities.

### Statistical analyses

#### Community analyses

To estimate chironomid species richness, we plotted species accumulation curves for each habitat with iNEXT [57] and tested for significant differences between habitat types by assessing the overlap of the 95% confidence intervals (CIs). We treated individual habitats as samples and used sample-based rarefaction curves standardized to sample coverages to compare species richness between habitat types [58]. Distance matrices were generated from the site-species data matrices using the Bray-Curtis metric [59]. Mantel tests were used to assess correlations among assemblage similarity matrices with the vegan package [60]. The species overlap between the reservoirs and the swamp forest was assessed using the number of shared species and the number of specimens for each shared species. Furthermore, the directionality of the species intermixing (e.g., reservoirs to the swamp forest or swamp forest to the reservoirs) was investigated by comparing the abundances of the shared species for each habitat.

#### Chironomid community structure in swamp forest

**Fig. 1:**
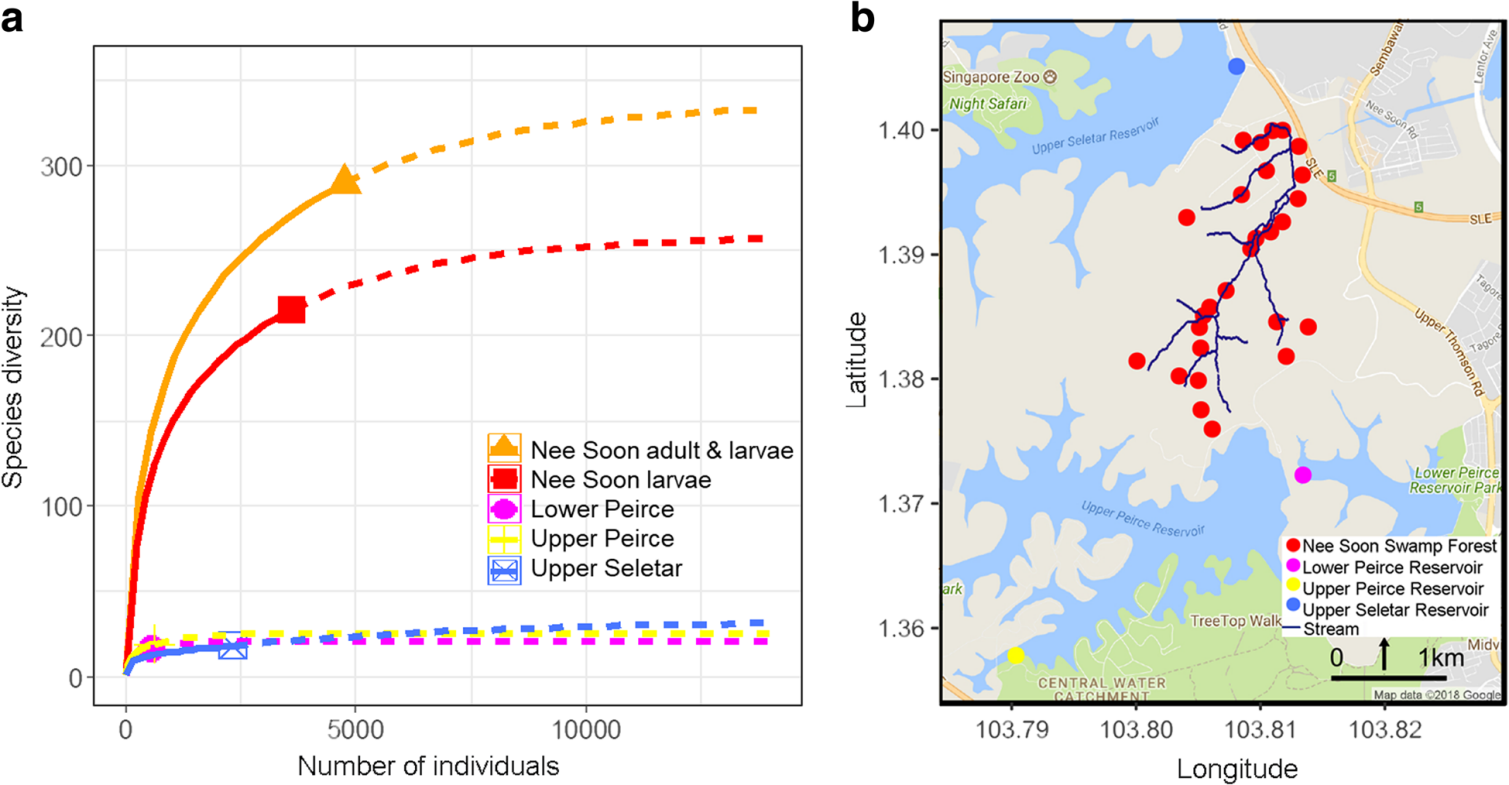
**a** Rarefaction curves (solid line) and extrapolation (dashed line) for chironomid communities of Nee Soon and reservoirs in Singapore. The 95% confidence intervals (shaded areas) were obtained by a bootstrap method based on 200 replications. **b** The distribution of the 28 sampling sites in the swamp forest and the three sampling sites in three reservoirs in the Central Catchment Region of Singapore. Different colors are given for each habitat. Stream lines were adopted from [102]

A multivariate approach (redundancy analysis, RDA) was used to assess whether there are important local variables that correlate with the chironomid community structure at the swamp forest sites (implemented using vegan package). The samples at each site were standardized to 70% sampling coverage (a measure of sampling completeness; see [61]) to minimize differences in abundance due to the different time/area sampled (see final analysis; Additional file 1: Table S1). As a result, only 28 of 40 sites were used for the following analysis (Fig. 1b). The species data matrix of 145 species in these sites was related to a total of 13 environmental (10 physicochemical, two spatial and one temporal) variables in RDA. Two variables (cross-sectional area and maximum depth) were removed from the analysis as they were highly correlated with stream width and average depth. All other predictor variables were tested for collinearity using variance inflation factor (VIF) function in R, but no VIF values larger than ten were found (see Additional file 2: Table S2). Thus they were retained. The statistical power of all analyses was assessed using a Monte Carlo permutation tests (*n* = 999).

#### Linear models

To assess the effects of environmental variables on species richness, evenness, and Shannon’s diversity in the swamp forest, linear mixed effect (LME) analysis was performed. This model was selected because it can account for non-independence of errors, i.e., due to spatial autocorrelation [62]. Spatial autocorrelation occurs when pairs of values, measured at given distances in space, are more similar than expected by chance alone [63]. Models with spatial correlation structures were generated using the corrSpatial argument in the nlme package [64]. Akaike information criterion (AIC) was used to compare the models. The model with the smallest AIC value was preferred. Hill numbers of order q: Species richness (*q* = 0), Shannon diversity (*q* = 1) and Simpson diversity (*q* = 2) were obtained with iNEXT. These values were used as dependent variables for three separate linear mixed-effects models [65] using the lme function with maximum likelihood estimation. For each model, continuous physicochemical variables and one categorical variable (presence-absence of the reservoir species) were used as fixed effects (without interaction term) nested within the sampling year as a random effect (see Additional file 2: Table S2). The categorical variable was used to test if reservoir species influenced the species richness in the swamp forest. Models were refined following the guidance in [66]: all parameters were included in the initial model with non-significant terms removed manually in a stepwise process, assessed by selecting the model with the lowest AIC value. If removal of a nonsignificant term increased the AIC value, the term was retained in the refined model. Once the final models were obtained, a linear model was fitted after removing random effects to assess the significance of each term in the model. The adjusted R^2^ value of the fitted model was calculated and compared with the adjusted R^2^ of models fitted with each parameter removed in turn. The relative contribution of each parameter in explaining the variance of the model was then calculated as a percentage of the total variance explained. *p* values for regression coefficients were obtained using the car package [67]. Statistics and graphical outputs were computed with the *ade4* package [68]. All statistical analyses were performed in R Version 3.4.0 [69] unless stated otherwise.

## Results

### Chironomid species richness at the reservoirs

In total, 33 species were observed in the reservoirs, and 61 ± 21 is the estimated species richness (Chao2). Across the three reservoirs, the most common chironomid species, *Polypedilum quasinubifer*, accounted for 48% of 3464 total chironomid specimens followed by *Polypedilum* sp. (near *leei*) (17%). The latter is likely to be a cryptic species related to *P. leei*, i.e., morphologically similar, however genetically more than 6% apart. The number of barcodes, sequenced specimens, and species for the individual reservoirs was as follows: Lower Peirce Reservoir: 544 of 1306; 17 observed species; 21 ± 5 estimated species; Upper Peirce Reservoir: 602 of 1056 specimens; 19 observed species; 25 ± 8 estimated species; Upper Seletar Reservoir: 2318 of 3647; 18 observed species; 33 ± 14 estimated species. The comparatively low barcoding success rate was due to sample handling (treatment with carbonated water and preservation in methylated ethanol).

### Chironomid species richness of the swamp forest

Based on a total of 6620 larval specimens sorted to Chironomidae, 4027 specimens were successfully barcoded (~ 61%). Of these, 417 were removed during the contamination check. Hence, a total of 3610 specimens were retained for further analysis (58.2%: 3610/6203). A total of 215 species was observed (estimated: 258 ± 16) with the proportion of singletons being high (23.2%) for the larval community of the Nee Soon Swamp Forest. Moreover, we barcoded 1551 adult specimens which yielded 1.278 sequences. After contamination check, 1141 adult specimens were retained for further analysis (81.1%: 1141/1414) and clustered into 158 putative species based on genetic distances (estimated: 214 ± 20). Singletons again represented a large proportion of the fauna (54 species, 34.2%), indicating the need for additional sampling. A total of 289 species were observed for the combined dataset of adult and larvae at Nee Soon Swamp Forest (n: 4751; estimated species richness: 336 ± 16). Adult and larval stages could be matched for 84 putative species.

### Stability of MOTU/species estimates for larval communities

The number of estimated MOTUs/species using the barcoding data was largely stable across a range of genetic distance thresholds: 227 (3%), 215 (4%), and 211 (5%). Most of the MOTUs were congruent (n: 197) between different thresholds, and the discrepancies were due to the assignment of 87 specimens lumping or splitting into different MOTUs depending on thresholds; i.e., the assignment of only 2% of the total number of specimens is sensitive to clustering thresholds. Given the stability of the results, we thus used MOTUs at 4% for all subsequent analyses. Most midge species were only found in Nee Soon Swamp Forest (207 of 240 species) while the observed chironomid richness in the three reservoirs was low as indicated by overlapping confidence intervals (see Fig. 1a).

### High species turnover between the reservoirs and the swamp forest

A total of 314 species was observed (estimated 371 ± 18) for the combined dataset of swamp forest and the reservoirs. However, the two habitats shared only eight species (Additional file 4: Table S3). Their overall community composition was not significantly correlated, based on an abundance dataset (NSSF - USR: Mantel *R* = − 0.03, NSSF - UP: *R* = − 0.02, NSSF - LP: R = − 0.02, *p* > 0.05 for all). Reservoirs shared more species with each other but only  the Lower Peirce and Upper Peirce reservoirs had significant albeit weakly correlated community composition (*R* = 0.19, *p* < 0.05) while they were dissimilar to Upper Seletar reservoir (LP - USR: *R* = − 0.07, UP - USR: *R* = − 0.11, *p* > 0.05 for both).

Of the final 28 sampling sites, only eight sites shared species with the reservoirs: seven sites each shared one species while one site (NS32, see Additional file 1: Table S1) shared six species. NS32 was relatively well sampled and is in close proximity to Upper Seletar Reservoir. We investigated the putative directionality of the species mixing (e.g., reservoirs to the swamp forest or swamp forest to the reservoirs) by comparing the abundances of the shared species in each habitat. We found that *Tanytarsus formosanus* had higher abundance in the swamp forest (82 specimens) than in the reservoirs (only four specimens) while the remaining six species were more common in the reservoirs and one species occurred in equal abundances in both habitats. All shared species had been previously recorded from the reservoirs in Singapore ([19, 20, 70] Baloğlu et al., unpublished). We hypothesized that those swamp forest sites sharing species with the reservoirs had overall lower species diversity than those without reservoir species. Using LME, we tested this hypothesis and found that there was no significant effect of the presence of reservoir species on the overall species richness, Simpson, and Shannon diversity indices (see Table 1).

**Table 1:** Linear mixed effects model to determine the relationships between three response variables (species richness, Shannon index, and Simpson index) in separate models and the continuous physicochemical variables and one categorical variable in 28 Nee Soon Swamp Forest sites

	Species richness		Shannon index		Simpson index	
Term	% Adj. R^2^	*P*	% Adj. R^2^	*P*	% Adj. R^2^	*P*
Conductivity	64.8	ns	69.8	*	78.8	*
Width	0	ns	0	ns	0	ns
Dissolved oxygen	15.6	ns	14.08	ns	6	ns
pH	0	ns	5.02	ns	15.2	ns
Presence of reservoir species	0	ns	0	ns	0	ns
Stream depth	0	–	0	–	0	–
Stream order	19.6	–	11.1	–	0	–
Stream discharge	0	–	0	–	0	–
Turbidity	0	–	0	–	0	–
Average velocity	0	–	0	–	0	–
Temperature	0	–	0	–	0	–
Total variance explained (Adj. R^2^)	0.12		0.27		0.17	

The relative contribution (%) of each term in explaining model variance was calculated as % difference in adjusted *R*^2^ comparing the full refined model and the model with each term removed. Stream depth, stream order, stream discharge, turbidity, average velocity, and the temperature were removed during model refinement. Symbols indicate the presence or the significance of the term within the refined model: 0, negative adjusted *R*^2^ values; −, not present in the refined model; ns, not significant, * = *P* < 0.05

### Habitat characteristics and chironomid species composition in the swamp forest

**Fig. 2:**
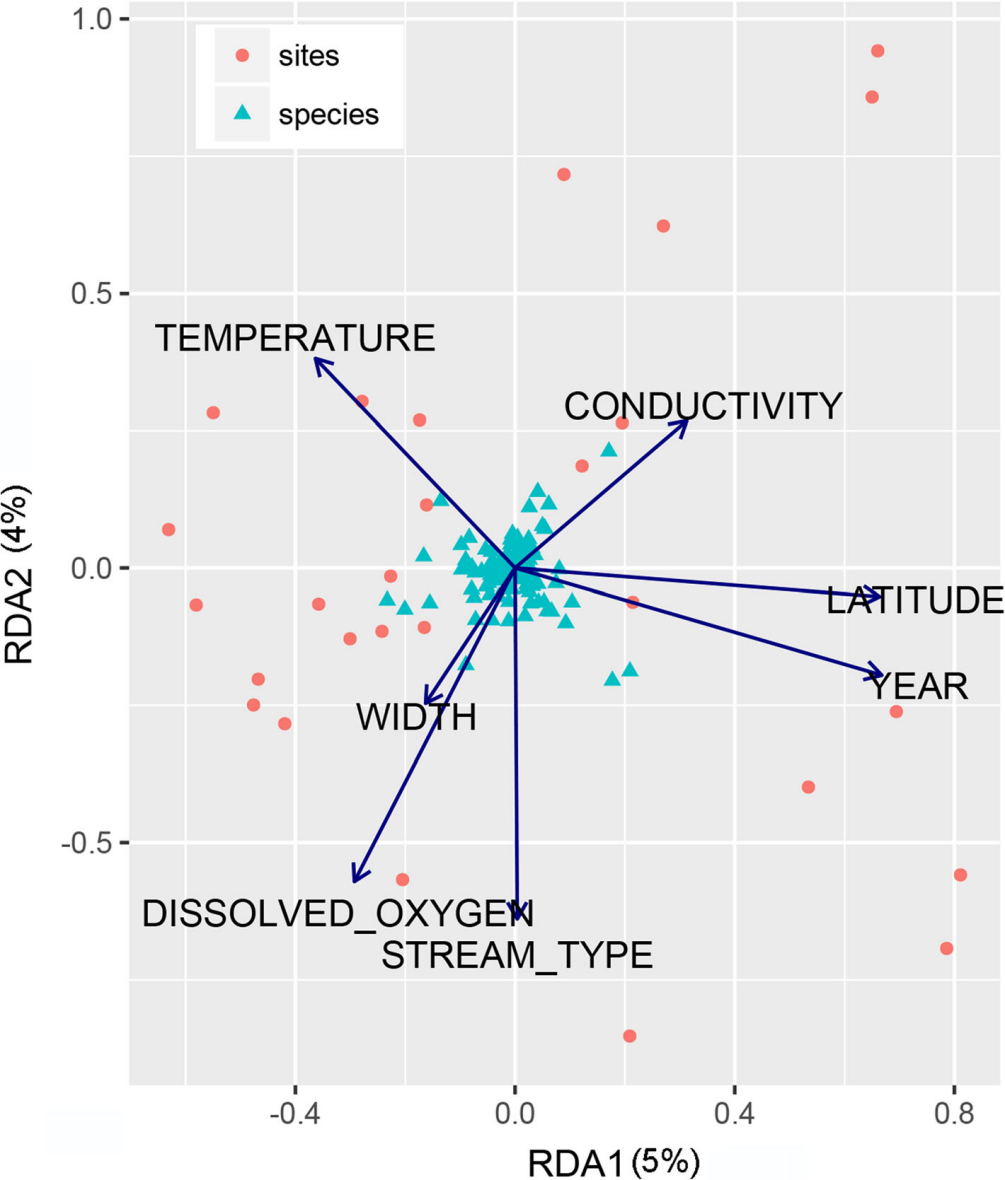
Ordination diagram from redundancy analysis (RDA) illustrating the relations between chironomid community composition and the environmental variables that explained the most variance. Solid arrows indicate the direction of sharpest increase in abundance of chironomid species

We found considerable variation in some of the environmental variables in Nee Soon Swamp Forest (see Additional file 2: Table S2). For instance, among physicochemical variables, water depth and turbidity ranged from 2.9 to 62.1 cm and from 0 to 1142.4 NTU, respectively. The first two axes of the RDA ordination analysis accounted for 64% of the total variance in the chironomid community composition, with the first axis explaining 26% of the variation and the Monte Carlo tests were significant for all axes, respectively (see Table 2 and Fig. 2).

**Table 2:** Weighted intraset correlation between the axes and the environmental variables following RDA of chironomid abundance data from Nee Soon Swamp Forest

	RDA1	RDA2	RDA3	RDA4
Eigen values	0.08	0.06	0.04	0.03
Accumulated % of the variance of species data explained	26	47	64	75
Correlation with axes				
Dissolved oxygen	−0.34	−0.68	−0.15	0.05
Stream order	0.005	−0.76	0.37	−0.08
Stream width	−0.19	−0.29	0.63	0.13
Temperature	−0.43	0.45	−0.25	−0.23
Conductivity	0.37	0.32	−0.21	−0.71
Latitude [flow direction]	0.78	−0.06	0.39	−0.1
Year	0.79	−0.23	−0.07	0.04

Only the significant variables are shown

All environmental variables combined explained 21% of the compositional variance. However, significant environmental (physicochemical, spatial, and temporal) variables selected by forward selection procedure explained only 18% of the compositional variance at Nee Soon (*F* = 1.80, *P* = 0.001). Dissolved oxygen levels, stream order, width, temperature, and the conductivity emerged as the most significant explanatory variables among the physicochemical variables (see Additional file 5: Table S4). Another significant variable was latitude which mostly represents flow direction from the upper to lower catchment. Variation partitioning analyses revealed that 10% of the total variance was explained by physicochemical variables alone, and 19% of the total variance was explained by all the variables (see Additional file 6: Table S5).

### What explains chironomid species richness in the swamp forest?

There was no evidence of spatial autocorrelation between the samples at different sites, as the AIC values of the models with spatial error structures were higher than the null models (data not shown). Therefore, the models without the spatial autocorrelation structure were selected. Statistical modeling (LME) was used to identify the dominant physicochemical variables in influencing the species richness, Shannon diversity, and Simpson diversity. We found that all three response variables were best predicted negatively by conductivity (i.e., ionic concentrations) with this term explaining most of the attributed variance in the model (Table 1), however, this was only significant for Shannon and Simpson diversity indices. Stream width, dissolved oxygen levels, pH, and the presence of reservoir species in the swamp forest were retained in the final models as non-significant terms, but only explained a small proportion of the variance.

## Discussion

### Impressive species richness of a tropical swamp forest remnant

Our study reveals a surprisingly species-rich chironomid community (336 estimated species) in the slow flowing streams of a relatively small (90 ha) remnant of a previously much larger swamp forest [71]. In order to fully appreciate these numbers, one should consider what is currently known about the global species diversity of Chironomidae. Different authors estimate that there are at least 10.000–20.000 species of which only approximately 5.000 have been described [72, 73]. However, our data imply that swamp forests can be so rich in midge diversity (nearly 350 species on 90 ha) that the global species estimates appear very conservative. After all, Nee Soon Swamp Forest is only a tiny remnant of an original lowland swamp forest [74] that was part of a more extensive freshwater swamp forest originally covering 5% of Singapore [71, 75]. Most of the world’s tropical swamp forests are found in Southeast Asia’s Indo-Malayan region (peat swamp forests: [76] and in the Amazon basin (freshwater swamp forests: [77]) and they collectively occupy a very large area (> 13 million ha; [78]) and are found on many geographically separated peninsulas and islands. Such biogeographic configurations tend to favor speciation. We propose that the chironomid midge diversity of swamp forests alone could exceed the lower bound estimates for global chironomid diversity. Unfortunately, much of this diversity is threatened with destruction, because especially the Southeast Asian peat swamps are disappearing fast [76] in the quest for more land for oil palm plantations and paper pulp production. For instance, more than half of the original peat swamp forest in Sumatra and Borneo have been converted to agriculture [79].

Our estimated chironomid species richness values exceed all values reported for chironomids in tropical streams, such as 299 species across 31 4th- to 6th-order West African streams, 250 species from 13 3rd- to 6th-order northwestern Costa Rican streams [80, 81], and 195 species from 15 1st- to 2nd-order streams in Brazil [82]. It has been suggested that the high richness values for tropical streams are mainly due to high numbers of rare species with very low abundances. This is also found in our study. A high proportion of species were only present at low abundances, and nearly half of the species were singletons. This implies that sampling has to be extensive and that specimen-based techniques such as NGS barcoding need to be used if most species are to be detected because bulk processing methods relying on metabarcoding struggle with detecting rare species based on the analysis of pooled DNA extractions.

Could it be that our results based on NGS barcodes overestimate species diversity? We believe that this is unlikely because several studies have documented high congruence between molecular and morpho-species for chironomids [26, 31, 73, 83]. In addition, our results are largely insensitive to which distance thresholds were used to estimate species numbers. For example, when we vary the clustering threshold from 3 to 5%, the corresponding species numbers only change from 327 to 309, i.e., overall stability at the MOTU level is at +/− 5%. Note that it is very likely that a large proportion of the species that were sequenced in this project are new to science (see Additional file 3 for taxa list) because only < 400 species of chironomid midges have been described for the Oriental region [84]. Note also that while the species numbers are likely to be only approximately correct, the species boundaries of a small number of MOTUs would likely change during taxonomic revision because DNA barcodes are likely to underestimate the species diversity of recently diverged species and overestimate species diversity for those species with diverging allopatric populations [85–89] because COI is not a speciation gene [90].

### Resistance of the swamp forest community to invasion from reservoirs

Our results suggest that the chironomid communities of both reservoirs and swamp forest are  very resistant to each other, i.e., their chironomid species richness and community composition are very different. Of the 215 species collected during the study from the larval communities, only eight species were found in the forest streams and reservoir habitats, signaling nearly complete community turnover within < 1 km. One could surmise that the resistance may be related to water pH differences between swamp forest and the reservoirs (see Additional file 2: Table S2). However, some nuisance midges are known to tolerate wide ranges of pH. For example, one of the species found in both habitats (*Tanytarsus formosanus*) is known from acidic rice fields in Malaysia (pH: 5.15**–**7.7 [91]: abundance positively correlated with pH). *Polypedilum leei*, another species that is found in both habitats has previously been reported to be present in acidic aquatic environments (pH: 4**–**7 [92], pH: 4**–**7.1 [93]). However, both *P. leei* and *P. quasinubifer* are widely distributed in Singapore’s reservoirs with neutral to alkaline water ([38], Baloğlu et al., unpublished). This means that pH alone is unlikely the only reason why few species are shared between the habitats.

With two exceptions species mixing was one-directional (reservoir to swamp forest; only exceptions are *Tanytarsus formosanus*: more common in the swamp forest and *Polypedilum leei*: equal abundance, see Additional file 4: Table S3). Yet, the shared species were found across several sampling sites in the swamp forest. This indicates that there was no major influence of the reservoirs on the adjacent swamp forest chironomid communities. Instead, it appears likely some chironomid adults are regularly blown to the different sampling sites, but only very few can establish temporary populations (note that we mostly processed larval midges). Due to the change in the direction of prevailing winds and the presence of both the Eastern and Western monsoon, no prediction can be made as to how wind will influence the direction of dispersal, but our results suggest that the overall integrity of Nee Soon’s midge fauna is secure with regard to invasion from urban reservoirs.

### Community patterns within Nee Soon Swamp Forest

Only a relatively small amount of the variance in midge community structure could be explained by the environmental parameters that were measured (~ 21%), but this may not be surprising given that no data were available for other variables known to be important such as food availability [94], species interactions, substrate [95], and the amount of vegetation cover [96]. Moreover, it is not atypical for studies of chironomid communities to find that abiotic factors explain a relatively small proportion of the variation (i.e., < 30%: [97, 98]). In our study, the most important physicochemical parameters were dissolved oxygen levels, stream order, width, temperature, and conductivity. This is in agreement with the previous studies [99, 100]. The changes in the latitude in the study are so small that the only spatial influence, “latitude” is here likely to reflect the direction of water flow from upper to lower catchment which may have some correlation with  stream order.

Conductivity (specific conductance) was negatively correlated with all three diversity indices but was significant for Shannon and Simpson diversity indices. Conductivity is here a measure of the concentrations of ions in the water. Nee Soon streams were reported to have low to medium conductivity (see Additional file 2: Table S2), indicating the poverty of nutrients and ionic concentrations in the water [101]. The uneven abundance of different species at the swamp forest may explain why only these two indices had a significant correlation, because these indices utilize both abundance and species richness data.

### Distribution of chironomid species within the swamp forest

We sampled adults at one sampling location and larvae at 40 sites. Overall, we were able to establish a larval-adult association for 84 putative species which illustrates the benefits of using cost-effective NGS barcodes on different life history stages [22]. However, it is likely that equal sampling would have increased the number of life stage matches. Overall, the larval sampling sites that were close to the adult sampling site were not likely to yield more associations. This may imply that adults disperse widely but may not lay eggs or may not be able to establish larval populations unless the environmental conditions are suitable. Given that some of the reservoir species were also found in Nee Soon, albeit in small abundances, the dispersal ability of chironomids may indeed not be the limiting factor for explaining why certain species are found in particular sites. It is more likely that the heterogeneity of the microhabitats is responsible for the species-rich and yet very complementary adjacent chironomid communities in the swamp forest.

### Effect of geography on chironomid distribution across reservoirs

Overall, we expected the three reservoirs to have similar chironomid communities because the physicochemical environments are similar based on a 13-year longitudinal study of environmental conditions. In addition, there is water flow from Upper Seletar to Lower Peirce and Upper Peirce Reservoirs [38]. However, according to a Mantel test, only the chironomid communities of the neighboring Lower and Upper Peirce reservoirs were very similar. The midge community of Upper Seletar Reservoir which is to the north of Nee Soon was more dissimilar despite the short distance between the reservoirs. It is conceivable that the swamp forest, with an environment that is apparently hostile to reservoir midges, is an effective barrier between the two similar reservoirs to the South of the swamp forest and the third reservoir to the North.

## Conclusions

Our results demonstrate that the tropical Nee Soon Swamp Forest has a surprisingly rich chironomid species diversity (~ 350 species) that is much higher than the diversity found in other tropical studies. Moreover, the swamp forest chironomid community is dramatically different from the community in surrounding reservoirs. Redundancy analyses and linear models suggest that the chironomid communities in the swamp forest were related to a mixture of physicochemical variables, such as dissolved oxygen levels, conductivity, stream order, width, and temperature but not to the distance between the sampling sites. However, the small amount of variance explained by these variables indicates that more environmental variables are needed for understanding the complex chironomid community structures in swamp forests. This study suggests that even fragmented or small swamp forest remnants, like the Nee Soon Swamp Forest, can be suitable habitats for a rich and likely native chironomid fauna. NGS barcoding was used in this study because it allows for processing large numbers of specimens. It can be easily adapted to other swamp forests in Southeast Asia for which no data are available. We thus hope that the results of this study will promote further studies of chironomid communities across Southeast Asia for characterizing and conserving the threatened fauna of Southeast Asian swamp forests.

## Additional files

Additional file 1: **Table S1**. Site name, code and location (geographical coordinates) for the study sites. Kick net sampling was used for Nee Soon Swamp Forest sites, and colonizer (UP, LP) and sediment grab (USR) were used for the reservoir sites. Final analysis indicates the sites which had at least 70% sampling coverage and were included in the analysis. (DOCX 22 kb)

Additional file 2: **Table S2**. Selected environmental characteristics and variance inflation factor (VIF) associated with each of the variables of 28 Nee Soon forest sites for redundancy analysis. Sampling method and device were also provided for each variable [103, 104]. (DOCX 16 kb)

Additional file 3: MOTU list. (XLSX 175 kb)

Additional file 4: **Table S3**. Shared species and their abundances between the Nee Soon Swamp Forest (adult and larvae) and reservoir chironomid communities. (DOCX 14 kb)

Additional file 5: **Table S4**. Results of a Monte Carlo test (999 permutations in the reduced model) for the redundancy analysis with a forward selection of environmental (physicochemical, spatial, and temporal) variables explaining the assemblage of chironomids in Nee Soon Swamp Forest. (DOCX 14 kb)

Additional file 6: **Table S5**. Variation partitioning results: Percentage of variation explained (pure and shared effect) for each group of variables classified by scale. (DOCX 13 kb)

## Abbreviations

COI: Cytochrome oxidase 1
LP: Lower Peirce
MOTU: Molecular operational taxonomic unit
NGS: Next generation sequencing
NSSF: Nee Soon Swamp Forest
RDA: Redundancy analysis
SE Asia: Southeast Asia
UP: Upper Peirce
USR: Upper Seletar Reservoir
