# Supplementary material for: NGS barcoding reveals high resistance of a hyperdiverse chironomid (Diptera) swamp fauna against invasion from adjacent freshwater reservoirs
Source: Front Zool. 2018 Aug 14;15:31. doi: 10.1186/s12983-018-0276-7 (PMC6092845; doi:10.1186/s12983-018-0276-7)
Supplement: Supplementary file 1 — Table S1. Site name, code and location (geographical coordinates) for the study sites. Kick net sampling was used for Nee Soon Swamp Forest sites, and colonizer (UP, LP) and sediment grab (USR) were used for the reservoir sites. Final analysis indicates the sites which had at least 70% sampling coverage and were included in the analysis. (DOCX 22 kb) [file 12983_2018_276_MOESM1_ESM.docx]

**Additional file 1 Table S1**. Site name, code and location (geographical coordinates) for the study sites. Kick net sampling was used for Nee Soon Swamp Forest sites and colonizer (UP, LP) and sediment grab (USR) was used for the reservoir sites. Final analysis indicates the sites which had at least 70% sampling coverage and were included in the analysis.

| **Site name** | **Site code** | |  | | **Latitude (°) decimal** | | **Longitude (°) decimal** | | **Sampling Date/Year** | | **Final**  **analysis** | |
| --- | --- | --- | --- | --- | --- | --- | --- | --- | --- | --- | --- | --- |
| Nee Soon | NS01 |  | | 1.37606 | | 103.80605 | | 20131008 | | ✓ | |  |
| Nee Soon | NS02 |  | | 1.37761 | | 103.80515 | | 20131014 | | ✓ | |  |
| Nee Soon | NS03 |  | | 1.37994 | | 103.80493 | | 20131018 | | ✓ | |  |
| Nee Soon | NS04 |  | | 1.37951 | | 103.80166 | | 20131018 | |  | |  |
| Nee Soon | NS05 |  | | 1.3803 | | 103.80339 | | 20131019 | | ✓ | |  |
| Nee Soon | NS06 |  | | 1.38149 | | 103.80500 | | 20131019 | |  | |  |
| Nee Soon | NS07 |  | | 1.38255 | | 103.80512 | | 20131101 | | ✓ | |  |
| Nee Soon | NS08 |  | | 1.38035 | | 103.79716 | | 20131101 | |  | |  |
| Nee Soon | NS09 |  | | 1.38151 | | 103.80002 | | 20131111 | | ✓ | |  |
| Nee Soon | NS10 |  | | 1.38326 | | 103.80240 | | 20131111 | |  | |  |
| Nee Soon | NS11 |  | | 1.38462 | | 103.80196 | | 20131122 | |  | |  |
| Nee Soon | NS12 |  | | 1.38379 | | 103.80410 | | 20131126 | |  | |  |
| Nee Soon | NS13 |  | | 1.38516 | | 103.80534 | | 20131128 | | ✓ | |  |
| Nee Soon | NS14 |  | | 1.38421 | | 103.80502 | | 20131128 | | ✓ | |  |
| Nee Soon | NS15 |  | | 1.38584 | | 103.80585 | | 20131130 | | ✓ | |  |
| Nee Soon | NS16 |  | | 1.38718 | | 103.80716 | | 20131204 | | ✓ | |  |
| Nee Soon | NS17 |  | | 1.38854 | | 103.80836 | | 20131204 | |  | |  |
| Nee Soon | NS18 |  | | 1.39054 | | 103.80915 | | 20131205 | | ✓ | |  |
| Nee Soon | NS19 |  | | 1.39136 | | 103.80956 | | 20131209 | | ✓ | |  |
| Nee Soon | NS20 |  | | 1.3919 | | 103.81075 | | 20131209 | | ✓ | |  |
| Nee Soon | NS21 |  | | 1.39454 | | 103.81295 | | 20131212 | | ✓ | |  |
| Nee Soon | NS22 |  | | 1.39645 | | 103.81330 | | 20131213 | | ✓ | |  |
| Nee Soon | NS23 |  | | 1.39677 | | 103.81324 | | 20131221 | |  | |  |
| Nee Soon | NS24 |  | | 1.39303 | | 103.80400 | | 20131221 | | ✓ | |  |
| Nee Soon | NS25 |  | | 1.39487 | | 103.80840 | | 20140114 | | ✓ | |  |
| Nee Soon | NS26 |  | | 1.3968 | | 103.81040 | | 20140114 | | ✓ | |  |
| Nee Soon | NS27 |  | | 1.39875 | | 103.81301 | | 20140116 | | ✓ | |  |
| Nee Soon | NS28 |  | | 1.39977 | | 103.81283 | | 20140116 | |  | |  |
| Nee Soon | NS29 |  | | 1.39925 | | 103.80854 | | 20140124 | | ✓ | |  |
| Nee Soon | NS30 |  | | 1.39907 | | 103.80996 | | 20140124 | | ✓ | |  |
| Nee Soon | NS31 |  | | 1.40002 | | 103.81090 | | 20140127 | | ✓ | |  |
| Nee Soon | NS32 |  | | 1.40005 | | 103.81171 | | 20140606 | | ✓ | |  |
| Nee Soon | NS33 |  | | 1.38188 | | 103.81200 | | 20140606 | | ✓ | |  |
| Nee Soon | NS34 |  | | 1.38425 | | 103.81377 | | 20140819 | | ✓ | |  |
| Nee Soon | NS35 |  | | 1.38346 | | 103.81187 | | 20140821 | |  | |  |
| Nee Soon | NS36 |  | | 1.38466 | | 103.81123 | | 20140905 | | ✓ | |  |
| Nee Soon | NS37 |  | | 1.38675 | | 103.81035 | | 20140917 | | ✓ | |  |
| Nee Soon | NS38 |  | | 1.38844 | | 103.80964 | | 20141114 | |  | |  |
| Nee Soon | NS39 |  | | 1.39029 | | 103.81012 | | 20141114 | |  | |  |
| Nee Soon | NS40 |  | | 1.39268 | | 103.81170 | | 20141212 | | ✓ | |  |
| Lower Peirce | LP |  | | 1.37236 | | 103.81335 | | 2014 | | ✓ | |  |
| Upper Peirce | UP |  | | 1.35787 | | 103.79027 | | 2014 | | ✓ | |  |
| Upper Seletar | USR |  | | 1.40516 | | 103.80802 | | 2013- 2014 | | ✓ | |  |
|  |  |  | |  | |  | |  | |  | |  |
|  |  |  | |  | |  | |  | |  | |  |
|  |  |  | |  | |  | |  | |  | |  |
|  |  |  | |  | |  | |  | |  | |  |
|  |  |  | |  | |  | |  | |  | |  |
|  |  |  | |  | |  | |  | |  | |  |
