# Supplementary material for: NGS barcoding reveals high resistance of a hyperdiverse chironomid (Diptera) swamp fauna against invasion from adjacent freshwater reservoirs
Source: Front Zool. 2018 Aug 14;15:31. doi: 10.1186/s12983-018-0276-7 (PMC6092845; doi:10.1186/s12983-018-0276-7)
Supplement: Supplementary file 2 — Table S2. Selected environmental characteristics and variance inflation factor (VIF) associated with each of the variables of 28 Nee Soon forest sites for redundancy analysis. Sampling method and device were also provided for each variable [103, 104]. (DOCX 16 kb) [file 12983_2018_276_MOESM2_ESM.docx]

**Additional file 2 Table S2.** Selected environmental characteristics and variance inflation factor (VIF) associated with each of the variables of 28 Nee Soon forest sites for redundancy analysis. Sampling method and device were also provided for each variable.

| **Variable** | **Abbreviation** | **Units** | **Min.** | **Max.** | **Median** | **VIF** | **Transformation** | **Method [reference]** |
| --- | --- | --- | --- | --- | --- | --- | --- | --- |
| Latitude | Lat | DD° | 1.37606 | 1.40005 |  | 4.49 | None | TRIMBLE GeoXH 6000 series GPS |
| Longitude | Long | DD° | 103.80339 | 103.81377 |  | 3.25 | None | TRIMBLE GeoXH 6000 series GPS |
| Year | Year |  | 2013 | 2014 |  | 2.79 | None |  |
| pH | pH | log[H+] | 3.83 | 5.88 | 4.44 | 3.66 | None | YSI 556 probe with YSI 5565A sensor |
| Specific conductance | Cond | mS cm^-1^ | 0.01 | 0.05 | 0.02 | 4.31 | None | YSI 556 probe with YSI 5560 sensor |
| Water depth | Av dep | cm | 2.9 | 62.1 | 14.5 | 4.08 | Log | [103] |
| Temperature | Temp | °C | 23.49 | 26.33 | 25.38 | 3.02 | Log | YSI 556 probe with YSI 5560 sensor |
| Dissolved oxygen | DO | mg L^-1^ | 0.33 | 6.62 | 5.71 | 2.85 | Log | YSI 556 probe with YSI 559 sensor |
| Width | Width | cm | 50 | 500 | 200 | 2.22 | Log | [103] |
| Average velocity | Av vel | cm s^-1^ | 1.1 | 16.5 | 6.1 | 2.49 | Log | HACH FH950 flow meter |
| Turbidity | Turb | NTU | 0 | 1142.4 | 16.1 | 1.5 | Log | Odeon turbidity meter and YSI 6600V2-4 probe |
| Stream discharge | Tot dis | m3 s^-1^ | 0 | 0.24 | 0.02 | 4.71 | None | [103] |
| Stream order | Strahler | None | 1 | 3 |  | 4.6 | None | [104] |
| Reservoir species presence in the swamp forest | non_nat | None | 0 | 1 |  |  | None |  |
