# Supplementary material for: NGS barcoding reveals high resistance of a hyperdiverse chironomid (Diptera) swamp fauna against invasion from adjacent freshwater reservoirs
Source: Front Zool. 2018 Aug 14;15:31. doi: 10.1186/s12983-018-0276-7 (PMC6092845; doi:10.1186/s12983-018-0276-7)
Supplement: Supplementary file 4 — Table S3. Shared species and their abundances between the Nee Soon Swamp Forest (adult and larvae) and reservoir chironomid communities. (DOCX 14 kb) [file 12983_2018_276_MOESM4_ESM.docx]

**Additional file 4 Table S3.** Shared species and their abundances between the Nee Soon Swamp Forest (adult and larvae) and reservoir chironomid communities.

|  | | **Sites/Communities** | | | | |  |  |
| --- | --- | --- | --- | --- | --- | --- | --- | --- |
| **Species** | | **Nee Soon larvae** | | **Nee Soon adults** | **Lower Peirce** | **Upper Peirce** | **Upper Seletar** | |
| *Ablabesmyia* typeTMSI | | 4 | | 0 | 12 | 15 | 0 | |
| *Cladotanytarsus* sp.4 | | 1 | | 0 | 4 | 0 | 140 | |
| *Polypedilum leei* | | 4 | | 0 | 2 | 1 | 1 | |
| *Polypedilum masudai* | | 0 | | 1 | 0 | 0 | 18 | |
| *Polypedilum quasinubifer* | | 2 | | 7 | 13 | 24 | 1628 | |
| *Tanytarsus formosanus* | | 82 | | 0 | 0 | 0 | 4 | |
| *Tanytarsus oscillans* | | 2 | | 1 | 0 | 2 | 42 | |
| *Tanytarsus ovatus* | | 23 | | 1 | 0 | 0 | 50 | |
