# Supplementary material for: NGS barcoding reveals high resistance of a hyperdiverse chironomid (Diptera) swamp fauna against invasion from adjacent freshwater reservoirs
Source: Front Zool. 2018 Aug 14;15:31. doi: 10.1186/s12983-018-0276-7 (PMC6092845; doi:10.1186/s12983-018-0276-7)
Supplement: Supplementary file 5 — Table S4. Results of a Monte Carlo test (999 permutations in the reduced model) for the redundancy analysis with a forward selection of environmental (physicochemical, spatial, and temporal) variables explaining the assemblage of chironomids in Nee Soon Swamp Forest. (DOCX 14 kb) [file 12983_2018_276_MOESM5_ESM.docx]

**Additional file 5 Table S4.** Results of a Monte Carlo test (999 permutations in the reduced model) for the redundancy analysis with forward selection of environmental (physicochemical, spatial, and temporal) variables explaining the assemblage of chironomids in Nee Soon Swamp Forest.

|  | **df** | ***F*-ratio** | ***P*-value** |  |
| --- | --- | --- | --- | --- |
| Stream order | 1 | 2.30 | 0.001*** |  |
| Stream width | 1 | 1.50 | 0.037* |  |
| Water temperature | 1 | 1.73 | 0.011* |  |
| Conductivity | 1 | 1.79 | 0 0.001*** |  |
| Dissolved oxygen levels | 1 | 1.81 | 0.004** |  |
| Latitude | 1 | 1.67 | 0.006** |  |
| Year | 1 | 1.83 | 0.003** |  |
