# Supplementary material for: NGS barcoding reveals high resistance of a hyperdiverse chironomid (Diptera) swamp fauna against invasion from adjacent freshwater reservoirs
Source: Front Zool. 2018 Aug 14;15:31. doi: 10.1186/s12983-018-0276-7 (PMC6092845; doi:10.1186/s12983-018-0276-7)
Supplement: Supplementary file 6 — Table S5. Variation partitioning results: Percentage of variation explained (pure and shared effect) for each group of variables classified by scale. (DOCX 13 kb) [file 12983_2018_276_MOESM6_ESM.docx]

**Additional file 6 Table S5.** Variation partitioning results: Percentage of variation explained (pure and shared effect) for each group of variables classified by scale

| **Effect** | **Adj. R^2^ (%)** |
| --- | --- |
| **Physicochemical, spatial, and temporal** | 19 |
| Physicochemical (Stream order, width, temperature, conductivity, dissolved oxygen levels) | 10 |
| Spatial (longitude, latitude) | 5 |
| Temporal (year) | 6 |
| Physicochemical*spatial | 0 |
| Temporal*spatial | 0 |
| Physicochemical*temporal*spatial | 2 |
|  |  |
